# Poly (ADP-Ribose) Polymerase-1 (PARP-1) Induction by Cocaine Is Post-Transcriptionally Regulated by miR-125b

**DOI:** 10.1523/ENEURO.0089-17.2017

**Published:** 2017-08-18

**Authors:** Sabyasachi Dash, Muthukumar Balasubramaniam, Tanu Rana, Arthur Godino, Emily G. Peck, Jeffery Shawn Goodwin, Fernando Villalta, Erin S. Calipari, Eric J. Nestler, Chandravanu Dash, Jui Pandhare

**Affiliations:** 1 Center for AIDS Health Disparities Research, Meharry Medical College, Nashville, TN 37208; 2 Center for Molecular and Behavioral Neuroscience, Meharry Medical College, Nashville, TN 37208; 3 School of Graduate Studies and Research, Meharry Medical College, Nashville, TN 37208; 4 Department of Biochemistry and Cancer Biology, Meharry Medical College, Nashville, TN 37208; 5 Department of Microbiology and Immunology, Meharry Medical College, Nashville, TN 37208; 6 Department of Pharmacology, Vanderbilt University Medical Center, Nashville, TN 37232; 7 Fishberg Department of Neuroscience and Friedman Brain Institute, Icahn School of Medicine at Mount Sinai, New York, NY 10029

**Keywords:** PARP-1, PARylation, cocaine, miRNA, post-transcriptional regulation

## Abstract

Cocaine exposure alters gene expression in the brain via methylation and acetylation of histones along with methylation of DNA. Recently, poly (ADP-ribose) polymerase-1 (PARP-1) catalyzed PARylation has been reported as an important regulator of cocaine-mediated gene expression. In this study, we report that the cellular microRNA “miR-125b” plays a key role for cocaine-induced PARP-1 expression. Acute and chronic cocaine exposure resulted in the downregulation of miR-125b concurrent with upregulation of PARP-1 in dopaminergic neuronal cells and nucleus accumbens (NAc) of mice but not in the medial prefrontal cortex (PFC) or ventral tegmental area (VTA). *In silico* analysis predicted a binding site of miR-125b in a conserved 3’-untranslated region (3’UTR) of the PARP-1 mRNA. Knockdown and overexpression studies showed that miR-125b levels negatively correlate with PARP-1 protein expression. Luciferase reporter assay using a vector containing the 3’UTR of PARP-1 mRNA confirmed regulation of PARP-1 by miR-125b. Specific nucleotide mutations within the binding site abrogated miR-125b’s regulatory effect on PARP-1 3’UTR. Finally, we established that downregulation of miR-125b and concurrent upregulation of PARP-1 is dependent on binding of cocaine to the dopamine transporter (DAT). Collectively, these results identify miR-125b as a post-transcriptional regulator of PARP-1 expression and establish a novel mechanism underlying the molecular effects of cocaine action.

## Significance Statement

Poly (ADP-ribose) polymerase-1 (PARP-1) plays critical roles in neuronal function. Recently, PARP-1 and PARylation have been described as a novel regulator of cocaine addiction and reward. However, the cellular pathways and mechanisms that regulate PARP-1 expression in neurons are unknown. We have identified that the cellular miRNA “miR-125b” negatively regulates PARP-1 protein expression. In addition, we have established that cocaine-induced upregulation of PARP-1 is dependent on miR-125b expression. These results elucidate the mechanism of PARP-1 regulation by cocaine and uncover a novel pathway for the cellular and molecular effects of cocaine.

## Introduction

Cocaine increases dopaminergic neurotransmission in key regions of brain and causes alterations in gene expression that play critical roles in reward and addiction (Ritz et al., 1987; Nestler, 2004; Kalivas, 2007). Cocaine exposure regulates gene expression by inducing chromatin remodeling via acetylation, methylation, and phosphorylation of histones and methylation of DNA (Chao and Nestler, 2004). Additionally, post-transcriptional regulation of gene expression by cellular microRNAs (miRNA) has also been reported for cocaine action (Picciotto, 2010; Eipper-Mains et al., 2011; Xu et al., 2013). Recently, poly ADP-ribosylation (PARylation) of chromatin by PAR polymerase-1 (PARP-1) has been described as a novel chemical modification for the long-term effects of cocaine (Scobie et al., 2014). PARP-1 catalyzes the synthesis of the negatively charged polymer PAR on specific amino acids of target proteins (Krishnakumar and Kraus, 2010). Accordingly, PARylation of histones has been shown to regulate gene expression via decondensation of chromatin (Fontan-Lozano et al., 2010; Verdone et al., 2015).

PARP-1 is a DNA damage sensor (Wei and Yu, 2016) and plays important roles in the normal function and diseased conditions of brain (Ha and Snyder, 2000; Cohen-Armon et al., 2004; Barr and Conley, 2007). PARP-1-mediated PARylation plays key roles in learning and memory (Cohen-Armon et al., 2004; Fontan-Lozano et al., 2010; Wang et al., 2012). Recently, it has also been reported that PARP-1 and PARylation are involved in cocaine-induced molecular, neural, and behavioral plasticity (Scobie et al., 2014). For example, chronic exposure to cocaine induced expression and activity of PARP-1 in the nucleus accumbens (NAc), a key brain reward region, of rodents. Importantly, cocaine-induced PARP-1 expression enhanced transcription of genes that are important in synaptic formation and dendritic plasticity (Scobie et al., 2014). These findings and others illustrate the important functions of PARP-1 and PARylation in addiction and reward. However, the mechanism and the cellular pathways that regulate PARP-1 in neurons are not identified. Several studies report transcriptional regulation of PARP-1 expression, since PARP-1 promoter sequences of human, mice and rat contain binding sites of transcriptional factors, including Sp1, YY1, AP-2, ETS, and NF-1 (Oei et al., 1997; Hassa and Hottiger, 1999; Zaniolo et al., 2007; Doetsch et al., 2012). Additionally, there are two reports suggesting post-transcriptional regulation of PARP-1 expression. In cancer cell models, the cellular miRNA “miR-124” has been shown to regulate PARP-1 (Chen et al., 2015), whereas miR-223 expression was negatively correlated with PARP-1 levels (Meloche et al., 2015). Surprisingly, regulation of PARP-1 gene expression in neuronal cells remains largely obscure.

We have identified miR-125b as a key post-transcriptional regulator of PARP-1 expression in neuronal cells. miR-125b is a highly conserved cellular miRNA (Lagos-Quintana et al., 2002) that regulates a network of genes involved in neuronal differentiation and function (Le et al., 2009). Our results show that acute and chronic cocaine exposure resulted in the downregulation of miR-125b concurrent with upregulation of PARP-1 in a dopaminergic neuronal cell model. Additionally, cocaine treatment resulted in the reduction in miR-125b levels and induction of PARP-1 in NAc of mice but not in medial prefrontal cortex (PFC) or ventral tegmental area (VTA). *In silico* analysis identified a binding site of miR-125b in the 3’-untranslated region (3’UTR) of PARP-1 mRNA that is conserved in various mammalian species. Knockdown and overexpression experiments demonstrate that PARP-1 expression is negatively correlated with miR-125b levels. Luciferase reporter assay show that miR-125b expression negatively regulates the activity of PARP-1 3’UTR and site directed mutagenesis confirm the direct binding of miR-125b to the PARP-1 3’UTR. Finally, we define that cocaine’s effect on miR-125b/PARP-1 axis is dependent on its binding to dopamine transporter (DAT). These studies establish that miR-125b is a key post-transcriptional regulator of PARP-1 in neurons.

## Materials and Methods

### Reagents

Cocaine hydrochloride, all-trans-retinoic acid (ATRA), and nomifensine were purchased from Sigma-Aldrich. Anti-PARP-1 and anti-caspase-9 antibodies were from Cell Signaling Technology, anti-DAT and anti-tyrosine hydroxylase (TH) antibodies were purchased from Abcam and anti-GAPDH and anti-β actin antibodies were procured from Sigma-Aldrich.

### Cell culture and cocaine treatment

Human neuroblastoma cell line (SH-SY5Y) was purchased from American type Culture Collection and was maintained in a 1:1 mixture of DMEM and Ham’s F12 medium (Gibco) supplemented with 10% (v/v) heat-inactivated fetal bovine serum (FBS; Gibco), 2 mM glutamine, and 1% antibiotics (penicillin-streptomycin) at 37°C in a humidified 5% CO_2_ atmosphere. For differentiation, the maintenance media was replaced with serum-free medium supplemented with ATRA at a final concentration of 10 μM and the cells were cultured for 4-6 d with change of media every alternate day. In all the experiments, appropriate number of SH-SY5Y cells were seeded and after differentiation, treated with cocaine in a dose-dependent manner from 1 μM to 100 μM. These concentrations were used based on published literature that highlight their physiologic relevance (Van Dyke et al., 1976; Mittleman and Wetli, 1984; Peretti et al., 1990; Karch et al., 1998; Blaho et al., 2000; Heard et al., 2008). The HEK-293T cells with stable expression of YFP-tagged DAT was grown and maintained in DMEM supplemented with 10% (v/v) heat-inactivated FBS, 2 mM glutamine, and 1% antibiotics (penicillin–streptomycin) at 37°C and 5% CO_2_. For acute treatment, cells were treated with cocaine overnight, whereas for chronic treatment, cells were exposed to cocaine once daily for 7 d.

### Phase contrast microscopy

SH-SY5Y cells were cultured and maintained in serum free-differentiation medium for 5 d on collagen coated six-well plates. Phenotypic changes were observed and monitored under bright field microscope. The differentiation was assessed by changes compatible with neuron-like morphology and neurite outgrowth from the cell body. Neurite extensions on captured images were measured using ImageJ 2.0 software (NIH) and lengths of individual neurites were calculated in pixels. Calculated values of neurite lengths were plotted as an average of field views for each sample (*n* = 10).

### Immunostaining and confocal microscopy

SH-SY5Y cells were seeded and differentiated on collagen coated glass coverslips. After differentiation the cells were fixed with 3.7% formaldehyde in PBS (1×) for 30 min, washed two times in PBS (1×) followed by blocking/permeabilization in a solution of PBS (1×) containing 10% FBS and 0.1% Triton X-100 for 30 min at room temperature. The cells were then stained at room temperature for 30 min with rat anti-DAT [1:200] and rabbit anti-TH (1:1500) prepared in blocking/permeabilization solution. Following incubation, the cells were washed three times with PBS (1×) for 5 min and then incubated with secondary antibodies such as chicken anti-rat IgG Alexa Fluor 488 conjugate and donkey anti-rabbit IgG Alexa Fluor 546, prepared in blocking/permeabilization solution, respectively, at room temperature for 30 min. The cells were then washed three times with PBS (1×) and mounted with ProLong Gold Antifade reagent with DAPI (Life Technologies) for nuclear staining on to glass slides. Confocal images were acquired using a Nikon TE2000-U laser-scanning confocal microscope, and data analysis was performed with NIS-Elements AR software (Nikon).

### Real-time PCR analysis

For measuring miRNA expression, total RNA was isolated by using miRNeasy mini kit (QIAGEN) as per manufacturer’s instructions. The cDNA was synthesized from the isolated total RNA using the miRCURY LNA Universal RT cDNA Synthesis kit (Exiqon). SYBR Green-based quantitative real-time PCR (qPCR) was used to quantify miR-125b or 5s ribosomal RNA (5srRNA) in a reaction mixture containing 50 ng of the cDNA as template and 300 nM LNA-based primers specific for miR-125b and 5srRNA (Exiqon). qPCR assay was performed in a C1000 Touch CFX96 system (Bio-Rad) as per manufacturer’s instructions. The expression levels (Ct values) of miR-125b are normalized to expression levels of 5s rRNA as ΔCt values. For multiple samples involving untreated control and treated samples, the relative expression levels of miR-125b is expressed as 2-Δ Ct values by comparing the ΔCt values of untreated control to the treated samples. Fold change in miR-125b expression was calculated by comparing the 2-Δ Ct values of the treated sample with that of untreated control.

For measuring PARP-1 mRNA expression, total RNA was isolated from untreated- and cocaine-treated cells and tissues using RNAesy Mini kit (QIAGEN) and cDNA synthesis was conducted using iScript cDNA synthesis kit (Bio-Rad). qPCR assay was performed by subjecting 50 ng of cDNA to iTaq Universal SYBR Green chemistry (Bio-Rad) using primers specific for PARP-1 (forward: 5’-GAGGTGGATGGGTTCTCTGA-3’ and reverse: 5’-ACACCCCTTGCACGTACTTC-3’) and GAPDH (forward: 5’-GAAGGTGAAGGTCGGAGTC-3’ and reverse: 5’-GAAGATGGTGATGGGATTTC-3’) as per manufacturer’s instructions. The expression levels of PARP-1 mRNA were normalized to that of GAPDH mRNA levels. Relative expression of PARP-1 mRNA in untreated control and treated samples is expressed as 2-Δ Ct values as described previously and fold changes are calculated by comparing the 2-Δ Ct values of the treated sample with that of untreated control.

### Bioinformatics analysis

Prediction of miR-125b binding sites in the 3’UTR of the *PARP-1* gene was conducted by submitting the miR-125b sequence and 3’UTR sequence of the *PARP-1* gene (Genbank accession number: NM_001618.3) to RNAhybrid 2.1 (Rehmsmeier et al., 2004) using default parameters. RNAhybrid analysis revealed three putative miR-125b binding sites in the PARP-1 3’UTR based on sequence match and predicted secondary structures. The putative targets obtained were further analyzed for sequence conservation across mammalian species using Clustal Omega multiple sequence alignment program (Sievers et al., 2011). The sequence with the highest identity (>90%) highlighted its functional significance and was chosen for further analyses in this study.

### Western blotting

SH-SY5Y cells were treated with various concentrations of cocaine for 24 h, after which the cells were harvested and washed with PBS (1×). Cell lysates were prepared using standard protocol and total protein concentrations were quantified by BCA protein assay (Pierce). Equal amounts of total protein from cell lysates were electrophoresed on SDS-polyacrylamide gels and transferred to nitrocellulose membranes using a semidry blotter (Bio-Rad). Membranes were blocked with 5% (w/v) nonfat milk in Tris-buffered saline (TBS; pH 8.0; Sigma). Blots were then probed with the primary antibody in blocking buffer, and subsequently by a secondary antibody conjugated to horseradish peroxidase (1:2000). All blots were washed in TBS with Tween 20 (pH 8.0; Sigma) and developed using the enhanced chemiluminescence (ECL) procedure (Pierce). Blots were routinely stripped by treating with Restore Plus stripping buffer (Pierce) and reprobed with anti-GAPDH or anti-β-actin monoclonal antibodies (Sigma) to serve as loading controls. Anti-rabbit antibody (Santa Cruz) was used as secondary antibody. Densitometry analyses were performed using LI-COR Image Studio version 5.2 software (LI-COR). Data were normalized to levels of GAPDH or β-actin.

### Flow cytometry

Neuronal apoptosis was determined by flow cytometry using the fluorescein isothiocyanate (FITC)–conjugated Annexin V apoptosis kit (Beckman Coulter) according to the manufacturer’s instructions. Untreated and cocaine-treated SH-SY5Y cells (1 × 10^6^) were washed in 1× annexin binding buffer and incubated for 15 min with Annexin V (AV)-FITC at 4°C. After two washes with binding buffer, propidium iodide (PI; 100 μg/mL) was added. Thereafter, cells were immediately analyzed by flow cytometry using FACSCalibur (Becton Dickinson). Positive staining of the plasma membrane with AV and lack of concomitant staining of nuclei with PI was measured as apoptosis at an early stage, whereas positive staining of cells with both AV and PI was indicative of apoptosis at later stages.

### Knockdown and overexpression of miR-125b in SH-SY5Y cells

miR-125b inhibitors/mimics and controls were purchased from GE-Dharmacon. Differentiated SH-SY5Y cells (2 × 10^5^ cells/well) grown in six-well culture plates were transfected with 100-300 pM anti-miRNAs/mimics or scrambled controls using Lipofectamine 3000 (Life Technologies). After transfection, cells were incubated for 48 h at 37°C/5% CO_2_, washed with PBS (1×), and harvested by gentle scraping for RNA and protein isolation.

### Luciferase assay

The luciferase reporter plasmid containing the full-length (763 nt) 3’UTR region of the PARP-1 gene (Genbank accession number: NM_001618.3) cloned downstream of the luciferase ORF in the pMirTarget vector (pPARP-3’UTR) was obtained from OriGene Technologies. To generate the control plasmid (pPARP-Null), the PARP-1 3’UTR insert was removed from pPARP-3’UTR by restriction digestion and the linearized plasmid was blunted using Klenow Polymerase (NEB) and subsequently religated using T4 DNA ligase (Promega). For mutating miR-125b binding site in the PARP-1 3’UTR (pPARP-3’UTRMut), we conducted site-directed mutagenesis using the QuikChange II XL Site-Directed Mutagenesis kit (Agilent Technologies) as per manufacturer’s protocol. Differentiated SH-SY5Y cells (4 × 10^4^ cells/well) in a 24-well plate were transfected with either the control plasmid without PARP-1 3’UTR (pmiRTarget) or plasmid harboring the full length PARP-1 3’UTR (pmiRTarget-UTR), or the pPARP3’UTRMut using Lipofectamine 3000 (Invitrogen) and incubated for 48 h. The transfection efficiency was measured by red fluorescence protein (RFP) expression. Transfected cells after treatment were lysed using 1× passive lysis buffer (Promega) and luciferase activity in the cell extracts was measured using a plate reader (BioTek). Samples were assayed in triplicate and the data are shown as luciferase activity normalized to RFP expression.

### Animals

All protocols were approved by the Institutional Animal Care and Use Committee at Icahn School of Medicine at Mount Sinai. For all experiments, 9- to 11-week-old C57BL/6J male mice (The Jackson Laboratory) were group housed (five per cage) in a colony room set at a constant temperature (23°C) on a 12/12 h light/dark cycle (lights on from 7 A.M. to 7 P.M.) with *ad libitum* access to food and water. For acute cocaine treatment the mice were injected (i.p.) with cocaine at 20 mg/kg body weight for 1 h while for chronic cocaine treatment the animals received daily i.p. injections for seven consecutive days of cocaine at 20 mg/kg unless otherwise stated. For time course experiments, animals were given daily injections (i.p.) of cocaine (20 mg/kg) and sacrificed on day 2, 4, or 6. Control mice for all groups received saline injections. Bilateral 14-gauge NAc punches were taken from each animal and frozen immediately.

### Isolation of total RNA, protein, and immunoprecipitation

Total RNA, including miRNA and protein were extracted from NAc, PFC or VTA punches using All-in-One kit (Norgen Biotek) as per the manufacturer’s instructions. The isolated total RNA was used to perform miRNA and PARP-1 mRNA expression analyses as described before. The extracted proteins were used for immunoprecipitation. Equal amount of protein extracts were mixed with 1 μl of anti-PARP-1 (46D11) monoclonal antibody (Cell Signaling) in 200 µl of IP buffer (50 mM HEPES, pH 7.5, 150 mM NaCl, 1.5 mM MgCl_2_, 1 mM EDTA, 2.5 mM EGTA, 0.1%Tween 20, 10% glycerol, and 1 mM DTT) containing protease inhibitors. The mixture was incubated with rotation at 4°C overnight. After which protein G-agarose suspension was added and further incubated with rotation at 4°C overnight. The beads were washed four times with IP buffer. Finally, the beads were resuspended in SDS sample buffer, heated at 95°C for 10 min, and briefly centrifuged. The proteins were separated by electrophoresis and detected by Western blotting.

### Statistical analysis

Data were expressed as mean ± SD obtained from three independent experiments. Significance of differences between control and treated samples was determined by Student’s *t* test. Values of *p* < 0.05 were considered statistically significant.

## Results

### Cocaine downregulates miR-125b expression in dopaminergic neuronal cell models and in the mouse NAc

**Figure 1.**
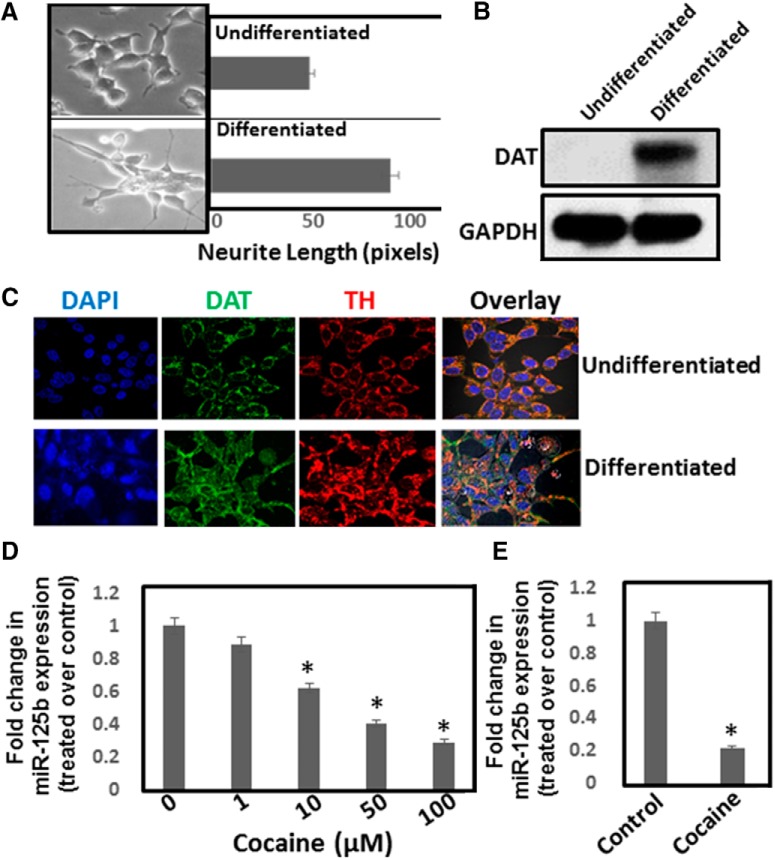
Cocaine exposure downregulates miR-125b expression. SH-SY5Y cells were cultured overnight and then differentiated for 3–5 d. ***A***, Dopaminergic phenotype of these cells was monitored via neurite length measurement by phase-contrast microscopy. ***B***, Western blot analysis of DAT expression in differentiated SH-SY5Y cells. ***C***, DAT and TH expression in differentiated SH-SY5Y cells were detected by confocal microscope. Data are representative of three independent experiments. ***D***, Differentiated SH-SY5Y cells were treated with varying concentrations of cocaine. Cells were harvested and miRNA expression was analyzed by qPCR. miR-125b expression was normalized to the 5S rRNA levels. Relative expression of miR-125b in cocaine-treated cells was compared to the untreated control cells and are plotted as fold change. ***E***, Mice were injected (i.p.) with a single dose of cocaine (20 mg/kg weight of mice) or saline and animals were sacrificed. NAc tissues were used for miRNA isolations and were subjected to qPCR. miR-125b expression was normalized to the 5s rRNA levels and relative expression of miR-125b in the NAc of cocaine-treated mice (*n* = 6) are expressed as fold changes to the NAc of saline-treated mice (*n* = 6). *p < 0.05 for the comparison of cocaine-treated samples versus untreated controls.

Earlier work in our laboratory showed that cocaine treatment resulted in the downregulation of miR-125b expression in lymphocytes (Mantri et al., 2012). Since miR-125b plays crucial roles in neuronal function (Le et al., 2009a and 2009b), we investigated the effects of cocaine exposure on miR-125b expression in neuronal cells. In initial studies, we used differentiated SH-SY5Y neuroblastoma cells (Fig. 1*A*) that express markers of dopaminergic neurons (Hartfield et al., 2014) such as DAT and TH (Fig. 1*B*,*C*). These cells were treated with varying concentrations of cocaine and miR-125b expression in these cells was measured by qPCR. Cocaine exposure resulted in the reduction of miR-125b expression in a dose-dependent manner relative to the untreated cells (Fig. 1*D*). Cells treated with 1 µM cocaine showed a minimal reduction in miR-125b expression, whereas with 100 µM cocaine, miR-125b level was reduced significantly when compared to the untreated cells (Fig. 1*D*).

Next, we examined the effects of cocaine exposure on miR-125b expression in a rodent model. Cocaine (20 mg/kg, i.p.) was administered to mice under acute conditions and the NAc, a key reward region of the brain, was isolated to measure miR-125b expression. qPCR analysis showed a significant reduction in miR-125b expression (∼80%) in the NAc of animals exposed to cocaine relative to the saline control animals (Fig. 1*E*). Collectively, the results in Figure 1 show that acute cocaine exposure downregulates miR-125b expression in a dopaminergic neuronal cell model and in the NAc of mice.

### Cocaine induces PARP-1 expression without eliciting neuronal apoptosis

**Figure 2.**
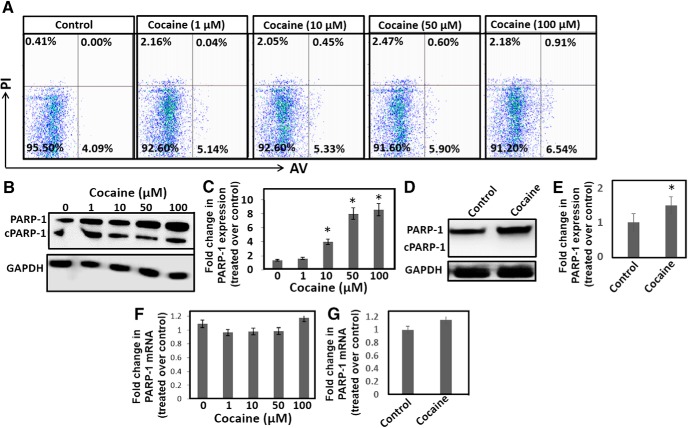
Cocaine exposure induces PARP-1 protein expression without increasing mRNA levels. ***A***, Differentiated SH-SY5Y cells were exposed to different concentrations of cocaine overnight and apoptosis was measured by flow cytometry using AV- as an early apoptosis marker and PI- as the late apoptotic marker. After cocaine treatment, cells were harvested and stained with FITC-conjugated AV/PI and analyzed by flow cytometry. Representative dot blot data showing percentage of cells stained positive for AV, PI, and AV + PI. ***B***, Differentiated SH-SY5Y cells were treated with varying concentrations of cocaine. Cells were harvested, and cell lysates were electrophoresed on denaturing acrylamide gels and transferred onto nitrocellulose membrane by electroblotting. Western blot analyses were performed using appropriate antibody and ECL kit. Representative data of PARP-1 expression in untreated and cocaine-treated cells. ***C***, Densitometry of PARP-1 expression from three independent experiments. ***D***, Mice were injected (i.p.) with a single dose of cocaine (20 mg/kg weight of mice, i.p.) or saline. Animals were sacrificed and NAc was isolated. Lysates prepared from the NAc tissues were subjected to western blot analysis. **(E)** Relative expression of PARP-1 in the NAc of cocaine-treated mice (*n* = 6) compared to the NAc of saline-treated mice (*n* = 6). *p < 0.05 for the comparison of cocaine-treated samples versus untreated controls. **(F)** Differentiated SH-SY5Y cells were treated with varying concentrations of cocaine overnight. Cells were harvested and total RNA was isolated. PARP-1 mRNA expression was measured by qPCR and was normalized to the levels of GAPDH mRNA. PARP-1 mRNA in cocaine-treated cells were expressed as fold changes over the untreated control cells based on ΔΔCt values. ***G***, Mice were injected (i.p.) with a single dose of cocaine (20 mg/kg weight of mice) or saline. Animals were sacrificed and NAc was isolated. Total RNA was isolated from the NAc tissues and were subjected to qPCR. PARP-1 mRNA expression was normalized to the GAPDH mRNA levels and fold changes in PARP-1 mRNA in the NAc of cocaine-treated mice (*n* = 6) over the NAc of saline-treated mice (*n* = 6) were calculated based on ΔΔCt values.

miR-125b negatively regulates p53 and a network of p53-induced genes that play critical roles in cellular apoptosis (Le et al., 2009a and 2009b). Therefore, we tested whether downregulation of miR-125b on cocaine treatment resulted in neuronal apoptosis. Differentiated SH-SY5Y cells were treated with increasing concentrations of cocaine and apoptosis was measured by flow cytometry using AV and PI staining. Surprisingly, cells treated with cocaine showed minimal increase in AV or PI staining compared to the untreated cells (Fig. 2*A*). Even in cells treated with the highest concentration of cocaine (100 µM), AV and PI staining remained relatively unchanged indicating minimal induction of neuronal apoptosis. We also measured expression of apoptotic markers such as caspase-9 and PARP-1 in cells treated with cocaine. Western blot analysis of the cellular lysates showed that cocaine treatment has no/minimal effect on the expression of caspase-9 or its cleaved forms (data not shown). Similarly, levels of cleaved PARP-1 remained unchanged in cells treated with or without cocaine (Fig. 2*B*), supporting the data in Figure 2*A* that cocaine exposure has minimal impact on neuronal apoptosis. Unpredictably, cocaine treatment resulted in a significant induction of total PARP-1 expression in these cells (Fig. 2*B*). Densitometry analysis showed a ∼eight to nine fold increase in total PARP-1 expression in cells treated with 100 μM cocaine compared to the untreated cells (Fig. 2*C*). Accordingly, induction of PARP-1 in the NAc of mice has been reported during chronic cocaine administration (Scobie et al., 2014). Therefore, we examined whether acute exposure of cocaine induced PARP-1 expression in the NAc of mice. Western blot analysis showed that PARP-1 expression was significantly induced in the NAc of mice treated with cocaine compared to the control animals (Fig. 2*D*,*E*). In addition, the levels of cleaved PARP-1 in the NAc was undetectable both in cocaine-treated and -untreated animals. The level of increased PARP-1 in NAc was consistent with the report by Scobie et al. (2014), albeit to a lower degree when compared to the neuronal cells. Nevertheless, results in Figure 2 suggest that cocaine-induced downregulation of miR-125b is not associated with neuronal apoptosis and induction of PARP-1 play important roles in the cellular and molecular effects of cocaine that is distinct from its reported role in cellular apoptosis.

### Cocaine induces PARP-1 expression through a post-transcriptional mechanism

PARP-1 expression is regulated transcriptionally by transcription factors Sp1, YY-1, and NF-κB (Oei et al., 1997; Hassa and Hottiger, 1999; Zaniolo et al., 2007; Doetsch et al., 2012). Thus, to test whether PARP-1-induction by cocaine is transcriptionally regulated, we quantified PARP-1 mRNA levels. Total mRNA from differentiated SH-SY5Y cells and the NAc tissues were subjected to qPCR. Interestingly, qPCR data revealed that PARP-1 mRNA levels were not significantly altered on cocaine treatment (Fig. 2*F*), although total PARP-1 protein levels were significantly increased in cocaine-treated cells (Fig. 2*B*). Similarly, minimal effect in the PARP-1 mRNA expression was observed in the NAc of cocaine-treated mice when compared to the saline-treated animals (Fig. 2*G*). These data suggest that cocaine-induced PARP-1 expression is not dependent on increased transcription and are consistent with the observation that transcriptional regulation does not sufficiently account for the increased PARP-1 expression by chronic cocaine exposure (Scobie et al., 2014).

### PARP-1 expression is post-transcriptionally regulated by miR-125b

**Figure 3.**
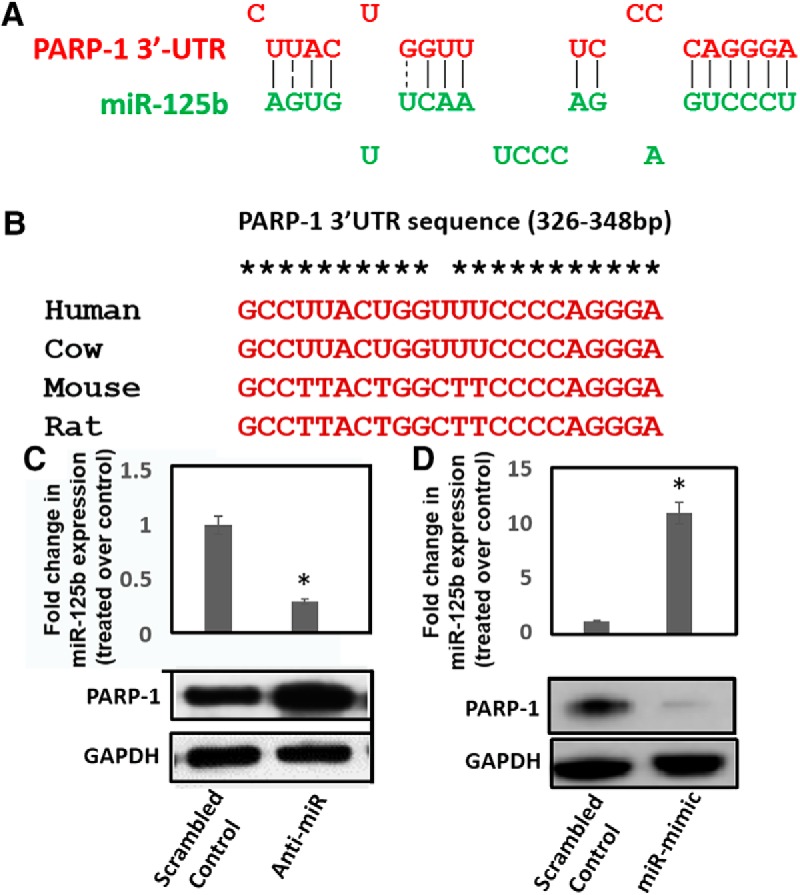
miR-125b expression negatively correlates with PARP-1 protein levels. ***A***, *In silico* analysis predicts putative binding site of miR-125b in the 3’UTR of PARP-1 mRNA. ***B***, Sequence alignment of miR-125b binding site in the 3’UTR of PARP-1 mRNA among different mammalian species. ***C***, upper panel, miR-125b knockdown. Chemically synthesized anti-miR-125b oligonucleotides were transfected into differentiated SH-SY5Y cells and reduction of miR-125b expression was measured 24 h post-transfection by qPCR. ***C***, lower panel, PARP-1 expression in miR-125b knocked-down cells was measured in the cellular lysates by immunoblot. ***D***, upper panel, miR-125b overexpression. Chemically synthesized miR-125b mimic oligonucleotides were transfected into differentiated SH-SY5Y cells and miR-125b expression was measured 24 h post-transfection by qPCR. ***D***, lower panel, PARP-1 expression in miR-125b overexpressing cells was measured in the cellular lysates by immunoblot. Data in ***C***, ***D*** are representative of three independent experiments. *p < 0.05 for the comparison of anti-miR-125b or miR-125b mimic samples versus scrambled controls.

Based on the data in Figure 2*F*,*G*, we envisioned that cocaine treatment induces PARP-1 expression via post-transcriptional mechanism. Although involvement of such mechanism in neuronal cells or by cocaine has not been previously reported, post-transcriptional regulation of PARP-1 has been documented in cancer cells (Schiewer and Knudsen, 2014). Interestingly, our data showed that cocaine exposure downregulated miR-125b levels (Fig. 1), concurrent with upregulation of PARP-1 expression (Fig. 2). Since miRNAs negatively regulate protein expression by binding to the 3’UTR of target mRNA (Bartel, 2009), we hypothesized that PARP-1 expression is post-transcriptionally regulated by miR-125b. In initial studies, we employed *in silico* analysis (Rehmsmeier et al., 2004) and identified three putative miR-125b binding sites at the 5’-end of the 3’UTR of PARP-1 mRNA (data not shown). Secondary structural analysis suggest that the site spanning the nucleotides 326-348 position of PARP-1 3’UTR is optimal for miR-125b binding (Fig. 3*A*). Importantly, this putative binding site, but not the other two, is evolutionarily conserved in the PARP-1 3’UTRs of several mammalian species (Fig. 3*B*). Thus, we tested a functional role of miR-125b in PARP-1 expression, by conducting knockdown and overexpression studies using anti-miR and miR-mimic oligonucleotides. Anti-miR-125b or miR-125b mimics were transfected into differentiated SH-SY5Y cells and expression of PARP-1 protein was measured in the cellular lysates by immunoblot. Results from these analyses showed that reducing miR-125b levels increased PARP-1 expression relative to that of the scrambled controls (Fig. 3*C*). Conversely, increasing miR-125b levels by miR-125b mimics markedly reduced PARP-1 protein levels (Fig. 3*D*). These data strongly suggested that miR-125b may serve as a negative regulator of PARP-1 expression in neuronal cells.

### MiR-125b binds to PARP-1 3’UTR to negatively regulate PARP-1 protein expression

**Figure 4.**
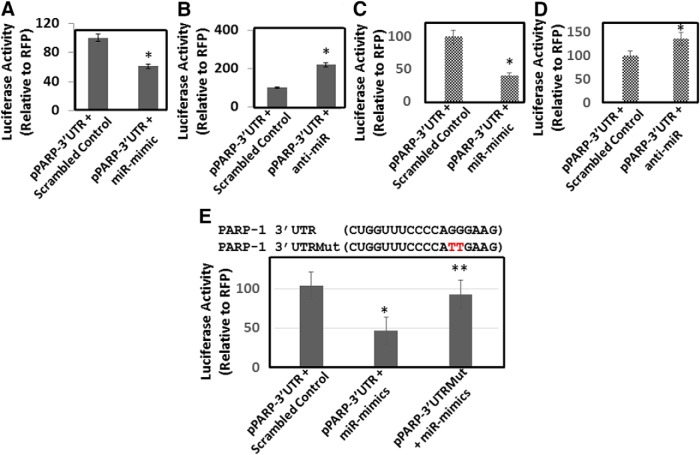
miR-125b targets 3’UTR of PARP-1 mRNA. We used a luciferase reporter vector, pPARP-3’UTR, containing the 3’UTR of PARP-1 mRNA cloned downstream of the luciferase stop codon. The pPARP-3’UTR was transfected into HEK-293T cells or differentiated SH-SY5Y cells in the presence or absence of miR-125b mimics or anti-miR-125b. ***A***, Luciferase activity of cellular lysates prepared from HEK-293T cells cotransfected with (pPARP-3’UTR) and scrambled control or miR-125b mimics. ***B***, Luciferase activity in cellular lysates prepared from HEK-293T cells cotransfected with (pPARP-3’UTR) and scrambled control or anti-miR-125b. ***C***, Luciferase activity in cellular lysates prepared from differentiated SH-SY5Y cells cotransfected with (pPARP-3’UTR) and scrambled control or miR-125b mimics. ***D***, Luciferase activity in cellular lysates prepared from differentiated SH-SY5Y cells cotransfected with (pPARP-3’UTR) and scrambled control or anti-miR-125b. ***E***, Site-directed mutagenesis of miR-125b binding site within the PARP-1 3’UTR. Upper panel, PARP 3’UTRMut represents mutation of two critical residues GG > TT. Lower panel, Luciferase activity in cellular lysates prepared from differentiated SH-SY5Y cells transfected with pPARP 3’UTR and pPARP 3’UTRMut in the presence or absence of miR-125b mimics. *p < 0.05 for the comparison of anti-miR-125b or miR-125b mimic samples versus scrambled controls. ***E***, **p < 0.05 for the comparison of miR-125b mimic samples of pPARP-3’UTR versus pPARP-3’UTRMut.

MiRNAs regulate protein translation by primarily binding to the 3’UTR of the target mRNA (Bartel, 2004; Jonas and Izaurralde, 2015; Lin and Gregory, 2015). Therefore, we examined whether miR-125b binds PARP-1 3’UTR through luciferase reporter assay. We used a vector containing the PARP-1 3’UTR downstream of the luciferase stop codon. The PARP-1 3’UTR construct (pPARP-3’UTR) or the control plasmid (pPARP-Null) was cotransfected into HEK-293T cells with or without miR-125b mimic or anti-miR-125b. Measurement of luciferase activity in the lysates of these cells showed that enhanced miR-125b expression significantly decreased luciferase activity relative to the control cells (Fig. 4*A*). Conversely, reducing miR-125b levels by transfection of anti-miR-125b resulted in a significant increase in luciferase activity (Fig. 4*B*). Since HEK-293T cells do not express endogenous DAT and lack the differentiated neuronal properties, we tested the effects of miR-125b expression on PARP-1 3’UTR activity in differentiated SH-SY5Y cells. Overexpression of miR-125b resulted in a significant reduction in luciferase activity (Fig. 4*C*), whereas knockdown studies of miR-125b enhanced luciferase activity (Fig. 4*D*). These data indicated a negative correlation between miR-125b expression and PARP-1 3’UTR driven luciferase activity.

To probe direct binding of miR-125b to PARP-1 3’UTR, we mutated specific nucleotides in the PARP-1 3’UTR sequence that are predicted to bind miR-125b (Fig. 4*E*). Then, we cotransfected pPARP-3’UTR or the control plasmid (pPARP-Null) in the presence of miR-125b mimic or miR-125b mutant into HEK-293T cells. As expected, luciferase activity measurements showed that overexpression of miR-125b reduced luciferase activity relative to the control cells (Fig. 4*E*). However, enhanced expression of miR-125b in cells cotransfected with the PARP-1 3’UTR mutant did not lead to reduction in luciferase activity (Fig. 4*E*). These data indicated that miR-125b negatively regulates PARP-1 by directly binding to the 3’UTR and support the hypothesis that PARP-1 is post-transcriptionally regulated by miR-125b in neuronal cells.

### Cocaine-induced downregulation of miR-125b is dependent on DAT

**Figure 5.**
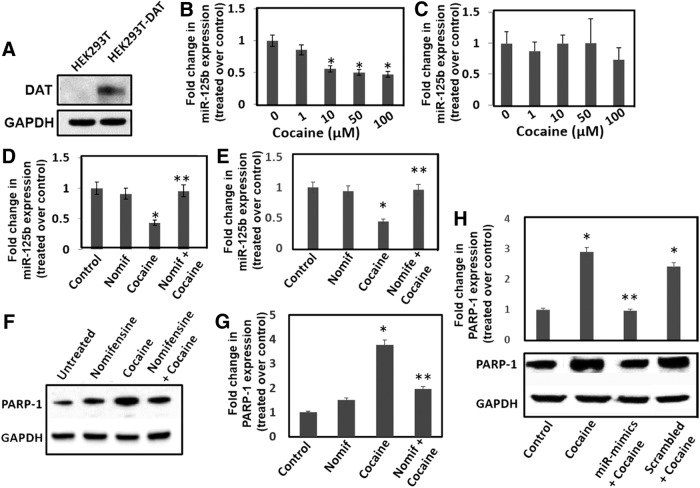
Cocaine-induced downregulation of miR-125b and concurrent upregulation of PARP-1 is dependent on DAT. ***A***, Detection of DAT in the HEK-293T-DATNull and HEK-293T-DAT cells by immunoblot. Fold change in miR-125b expression in (***B***) HEK-293T-DAT cells and (***C***) HEK-293T-DAT-Null cells on exposure to varying concentration of cocaine relative to untreated cells as measured by qPCR. ***D***, Fold change in miR-125b expression in HEK-293T-DAT cells pretreated with nomifensine. ***E***, ***G***, Differentiated SH-SY5Y cells with or without nomifensine pretreatment were treated with cocaine. ***E***, miR-125b expression was measured in these cells by qPCR. ***F***, PARP-1 expression was measured in the cell lysates by Western blotting. ***G***, Densitometry of PARP-1 expression from three independent experiments. ***H***, Differentiated SH-SY5Y cells were transfected with miR-125b mimics or scrambled controls and then treated with cocaine (50 μM) overnight. PARP-1 expression was measured in the lysates of these cells by immunoblot. Lower panel shows representative data, whereas upper panel shows fold change in PARP-1 expression from three independent experiments. *p < 0.05 for the comparison of cocaine-treated samples versus untreated controls. ***D***, ***E***, ***G***, **p < 0.05 for the comparison of cocaine-treated samples versus nomifensine + cocaine-treated samples. ***H***, **p < 0.05 for the comparison of cocaine-treated samples versus miR-mimic + cocaine-treated samples.

DAT is well established to play critical roles in the cellular and molecular effects of cocaine addiction and reward (Kuhar et al., 1991). Thus, we examined whether binding of cocaine to DAT is key for the downregulation of miR-125b by using DAT-expressing and DAT-null HEK-293T cells (Fig. 5*A*). These cells serve as appropriate models since they endogenously express miR-125b (Fig. 5*B*,*C*). Exposure of DAT-expressing cells to increasing concentrations of cocaine resulted in a dose-dependent reduction in miR-125b levels (Fig. 5*B*), mirroring the observations with dopaminergic neuronal cells and animal studies (Fig. 1). Interestingly, in DAT-null cells, cocaine exposure did not result in the reduction of miR-125b expression even with the highest dose of cocaine (Fig. 5*C*), suggesting that DAT plays a critical role in the downregulation of miR-125b expression. To further probe the involvement of DAT, we used “nomifensine,” a DAT blocker and dopamine uptake inhibitor (Ferris et al., 2012). Nomifensine treatment alone did not alter miR-125b expression in DAT-expressing cells (Fig. 5*D*). Remarkably, pretreatment of DAT-expressing cells with nomifensine before cocaine treatment significantly abrogated cocaine-induced reduction in miR-125b expression (Fig. 5*D*). Next, we performed the nomifensine pretreatment studies in differentiated SH-SY5Y cells as a model for dopaminergic neurons. Similar to the data in Figure 5*D*, nomifensine treatment did not change miR-125b expression in these cells. However, pretreatment of cells with nomifensine markedly reduced cocaine’s effect on miR-125b expression (Fig. 5*E*). Collectively, these data provide strong evidence that miR-125b downregulation is dependent on the binding of cocaine to DAT.

### Binding of cocaine to DAT regulates miR-125b/PARP-1 axis

Our studies with the dopaminergic neuronal cells illustrated that miR-125b expression negatively regulates PARP-1 expression (Fig. 3). Data in Figure 5*B*,*C* showed that downregulation of miR-125b expression is dependent on binding of cocaine to DAT. Based on these observations, we hypothesized that cocaine exposure activates miR-125b mediated regulation of PARP-1 by binding to DAT and inhibition of DAT binding abrogates cocaine’s effect on miR-125b/PARP-1 axis. To test this, we treated differentiated SH-SY5Y cells with cocaine and asked whether pretreatment of nomifensine affected cocaine-induced PARP-1 expression. Lysates of cells exposed to cocaine with or without nomifensine pretreatment were subjected to Western blot analysis. As expected cocaine treatment increased PARP-1 expression, whereas nomifensine treatment did not alter PARP-1 levels (Fig. 5*F*,*G*). However, cocaine-induced PARP-1 expression was abrogated in cells that were pretreated with the inhibitor (Fig. 5*F*,*G*). These data provided further evidence that cocaine-induced PARP-1 expression and the concurrent miR-125b downregulation is primarily driven by direct binding of cocaine to DAT. Finally, we tested whether overexpression of miR-125b abrogates cocaine’s effect on PARP-1 expression. miR-125b mimics were transfected into differentiated SH-SY5Y cells and overexpression of miR-125b was confirmed by qPCR (data not shown). Then, these cells were treated with cocaine (50 µM) overnight and PARP-1 expression was measured by immunoblot. Results in Figure 5*H* show that cocaine treatment induced PARP-1 expression in control cells (lane 2 and lane 4). However, miR-125b overexpression inhibited induction of PARP-1 on cocaine treatment (lane 3). These observation strongly support the hypothesis that cocaine’s effect on PARP-1 expression is regulation by miR-125b

### Chronic cocaine exposure activates miR-125b/PARP-1 axis

**Figure 6.**
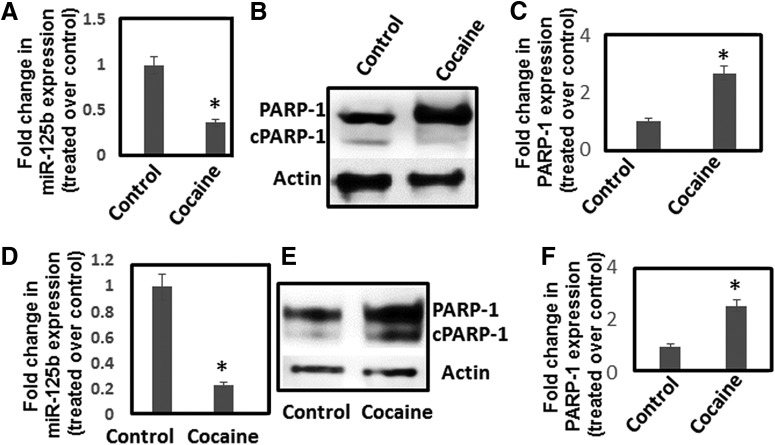
Chronic cocaine exposure reduces miR-125b expression and upregulates PARP-1 expression. ***A–C***, Differentiated SH-SY5Y cells were treated daily with varying concentrations of cocaine for 7 d. Cells were harvested, and miR-125b expression was measured by qPCR (***A***), whereas PARP-1 expression was detected by Western blotting (***B***, ***C***). ***A***, miR-125b expression was normalized to 5S rRNA levels and plotted as fold changes in miR-125b expression in cocaine-treated cells relative to untreated control. ***B***, Representative immunoblot of PARP-1 expression in control and cocaine-treated cells. ***C***, Relative PARP-1 expression in cocaine-treated cells and untreated cells as quantified by densitometry from three independent experiments. ***D-F***, Mice were injected (i.p.) with cocaine (20 mg/kg weight of mice) or saline for 7 d. Thereafter, animals were sacrificed and NAc was isolated. RNA and lysates from the NAc tissues were subjected to qPCR and Western blotting, respectively. ***D***, miR-125b expression was normalized to the 5s rRNA levels and relative expression of miR-125b in the NAc of cocaine-treated mice (*n* = 6) compared to the NAc of saline-treated mice (*n* = 6) are expressed as fold changes. ***E***, Representative immunoblot showingPARP-1 expression in the NAc of cocaine-treated mice and saline-treated control mice. ***F***, Relative expression of PARP-1 in the NAc of cocaine-treated mice (*n* = 6) compared to the NAc of saline-treated control mice (*n* = 6) based on densitometry analysis. *p < 0.05 for the comparison of cocaine-treated samples versus untreated controls.

Our results showed that acute cocaine exposure downregulates miR-125b leading to the upregulation of PARP-1. It has also been reported that chronic cocaine exposure upregulates PARP-1 expression in NAc of mice (Scobie et al., 2014). Therefore, we examined whether cocaine targets miR-125b under chronic exposure conditions both *in vitro* and *in vivo*. Differentiated SH-SY5Y cells were exposed to cocaine (50 μM) for 7 d. To reduce degradation of cocaine in the culture media, we used heat-inactivated serum in the media. qPCR analysis showed that chronic cocaine exposure resulted in a dose-dependent reduction in miR-125b expression (Fig. 6*A*). In addition, Western blot analysis illustrated increased PARP-1 levels in cocaine exposed neuronal cells without significant change in the cleaved form (Fig. 6*B*,*C*). Similarly, cocaine was administered to mice under chronic conditions (7 d with one dose daily) and NAc regions of mice either treated with cocaine or saline were isolated. Total RNA and protein were isolated from NAc for analyses of miR-125b and PARP-1 expression, respectively. We observed that, similar to the acute cocaine treatment, chronic cocaine exposure significantly downregulated miR-125b expression in NAc compared to saline-treated mice (Fig. 6*D*). Moreover, we also observed increased PARP-1 protein levels in the NAc of mice exposed to chronic cocaine treatment albeit accompanied by a slight increase in cleaved PARP-1 level (Fig. 6*E*,*F*).

**Figure 7.**
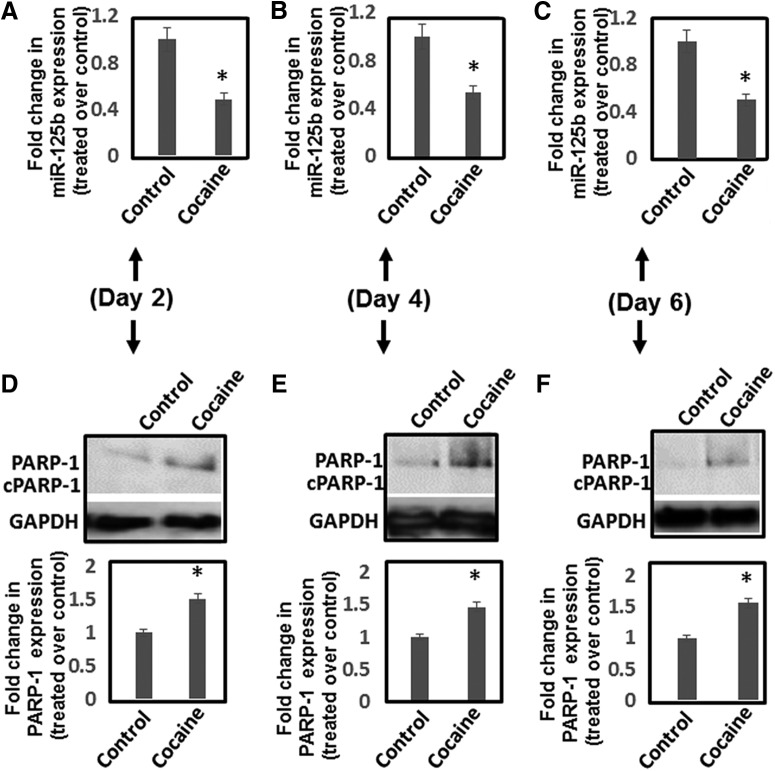
Time-dependent effects of cocaine exposure on miR-125b and PARP-1 expression in NAc. Mice were injected (i.p.) with cocaine (20 mg/kg weight of mice) or saline for 2, 4, and 6 d. Thereafter, animals were sacrificed, and NAc regions were isolated. RNA and lysates from the NAc tissues were subjected to qPCR and Western blotting for measuring miR-125b and PARP-1 expression, respectively. ***A–C***, Fold changes in miR-125b expression in cocaine-treated (n = 6) and saline-treated control (n = 6) animals on (***A***) day 2, (***B***) day 4, and (***C***) day 6. ***D–F*,** PARP-1 protein expression in the NAc tissues was analyzed by immunoblot and subjected to quantification by densitometry. Upper panels show representative data of PARP-1, whereas lower panels show densitometry of PARP-1 expression from cocaine-treated mice (*n* = 6) and saline-treated control animals (*n* = 6). Fold changes in PARP-1 expression in cocaine-treated mice and saline-treated controls as measured by densitometry of immunoblots of samples on (***D***) day 2, (***E***) day 4, and (***F***) day 6. *p < 0.05 for the comparison of cocaine-treated samples versus untreated controls.

To better understand the effects of cocaine exposure on miR-125b and PARP-1 expression, we conducted time course analysis of miR-125b downregulation and PARP-1 induction on cocaine treatment. Animals were given daily injections of cocaine (20 mg/kg) and sacrificed on day 2, 4, and 6. NAc was isolated and analyzed for miR-125b and PARP-1 expression. qPCR data showed that miR-125b expression remained downregulated throughout the 6 d of cocaine administration (Fig. 7*A–C*). Immunoblot analysis revealed that PARP-1 expression was consistently upregulated during treatment period (Fig. 7*D-F*). Taken together, these results illustrate that cocaine-induced downregulation of miR-125b is associated with increased levels of PARP-1 expression both under acute and chronic conditions.

### Effects of cocaine exposure on miR-125b and PARP-1 in the VTA and PFC

**Figure 8.**
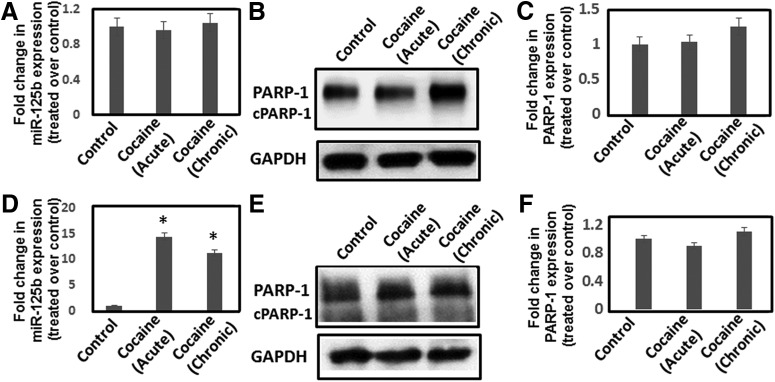
Effects of cocaine exposure on miR-125b and PARP-1 expression in PFC and VTA. Mice were injected (i.p.) with cocaine (20 mg/kg weight of mice) or saline under acute and chronic conditions. Thereafter, animals were sacrificed and PFC (***A–C***) and VTA (***D–F***) were isolated. RNA and lysates from the tissues were subjected to qPCR and Western blotting for measuring miR-125b and PARP-1 expression, respectively. Fold changes in miR-125b expression in (***A***) the PFC and (***D***) the VTA of cocaine-treated animals and saline-treated control animals were calculated. PARP-1 expression in (***B***) the PFC and (***E***) the VTA of cocaine-treated mice and saline-treated controls as measured by immunoblot. Fold changes in PARP-1 protein expression in (***C***) the PFC and (***F***) the VTA of cocaine-treated mice (*n* = 6) compared to saline-treated control mice (*n* = 6). *p < 0.05 for the comparison of cocaine-treated samples versus untreated controls.

To examine whether cocaine regulates miR-125b and PARP-1 expression specifically in the NAc, we tested the effects of cocaine in other regions of the brain’s reward pathway including VTA and PFC. Cocaine (20 mg/kg) was administered to mice under acute and chronic conditions and the VTA and PFC were isolated. RNA and protein preparations of these tissues were subjected to qPCR and immunoblot analysis to measure miR-125b and PARP-1 expression, respectively. Data from these analysis revealed that both acute and chronic cocaine treatment minimally affected miR-125b expression (Fig. 8*A*) and PARP-1 expression (Fig. 8*B*,*C*) in PFC. However, in the VTA, while PARP-1 expression was not affected (Fig. 8*E*,*F*), miR-125b levels were significantly elevated by acute and chronic cocaine treatment (Fig. 8*D*). These results suggest that miR-125b levels is not negatively correlated with PARP-1 expression in the VTA. Still, these results show that a marked effect on the downregulation of miR-125b and PARP-1 induction is largely restricted to the NAc after acute and chronic cocaine exposure.

## Discussion

Cocaine-induced alterations in cellular miRNA expression and subsequent post-transcriptional regulation of gene expression in specific brain regions are well documented (Hollander et al., 2010; Chen et al., 2013; Xu et al., 2013; Doura and Unterwald, 2016). In the mesolimbic dopaminergic system, chronic cocaine administration downregulates expression of let-7d and miR-124 while upregulating miR-181a (Chandrasekar and Dreyer, 2009; Chandrasekar and Dreyer, 2011), Accordingly, in the dorsal striatum, cocaine exposure upregulates miR-212 along with its homolog miR-132 (Im et al., 2010). In the hippocampus, cocaine upregulates miR-181a (Chandrasekar and Dreyer, 2009) and several other miRNAs, including miR-134, miR-152, and miR-26b (Chen et al., 2013). A set of miRNAs were also shown to be specifically modulated in the striatal post-synaptic densities (PSDs; Eipper-Mains et al., 2011). Altered expression of these cellular miRNAs has been demonstrated to play functional roles in cocaine reward and addiction. Results described in this report provide evidence that miR-125b is important for cocaine’s action in the brain.

MiR-125b is highly expressed in the mammalian brain and regulates several key functions including neuron survival, differentiation, and synaptic plasticity (Lagos-Quintana et al., 2002; Le et al., 2009). Previous work from our laboratory has demonstrated that cocaine downregulates miR-125b expression in human CD4+ T cells (Mantri et al., 2012). In this study, we report that both acute and chronic cocaine exposure downregulates miR-125b expression in an *in vitro* model of dopaminergic neuronal cells and *in vivo* in the NAc region of mice (Fig. 1). Although downregulation of miR-125b is well established to activate cellular apoptotic pathway (Le et al., 2009a and 2009b), exposure of cocaine did not induce neuronal apoptosis as measured by AV/PI staining and cleavage of PARP-1 (Fig. 2). Surprisingly, a significant increase in the total PARP-1 protein was observed in response to cocaine exposure both *in vitro* (Fig. 2) and *in vivo* (Fig. 6). These results are in accordance with the reported induction of PARP-1 protein levels in NAc on chronic cocaine administration (Scobie et al., 2014).

PARP-1 is a highly conserved nuclear enzyme that plays a central role in cellular stress response, DNA damage, and repair (Lupo and Trusolino, 2014; Morales et al., 2014; Schiewer and Knudsen, 2014). PARP-1 also regulates cellular gene expression (Fontan-Lozano et al., 2010; Verdone et al., 2015), since activation of PARP-1 results in the addition of negatively charged PAR (aka PARylation) to histones that induce chromatin remodeling (Krishnakumar and Kraus, 2010; Wei and Yu, 2016). Because of these essential roles, PARP-1 is also involved in neuronal function, learning and memory (Ha and Snyder, 2000; Cohen-Armon et al., 2004; Fontan-Lozano et al., 2010; Wang et al., 2012). For example, recent evidence suggests that PARP-1 and PARylation play crucial roles in cocaine induced molecular, neural, and behavioral plasticity (Scobie et al., 2014; Scobie, 2015). Yet, the cellular mechanisms that regulate PARP-1 expression are poorly defined. There is evidence that PARP-1 expression is transcriptionally regulated by transcriptional factors such as Sp1, YY1, AP-2, ETS, and NF-1 (Oei et al., 1997; Hassa and Hottiger, 1999; Zaniolo et al., 2007; Doetsch et al., 2012). Accordingly, *in silico* studies of the PARP-1 promoters of several mammalian species predicted potential binding sites of several other transcriptional factors (Oei et al., 1997; Hassa and Hottiger, 1999; Zaniolo et al., 2007; Doetsch et al., 2012). Interestingly, in cancer cells, PARP-1 expression has been reported to be post-transcriptionally regulated by miR-124 (Chen et al., 2015). Similarly, a negative correlation between miR-223 expression and PARP-1 mRNA levels was also observed (Meloche et al., 2015). However, the cellular and molecular determinants that regulate PARP-1 expression in neurons are largely unknown.

We provide strong evidence that PARP-1 is post-transcriptionally regulated by miR-125b in neuronal cells. We observed that PARP-1 induction is correlated with reduction in miR-125b expression both in dopaminergic neuronal cultures and NAc of mice by acute and chronic cocaine exposure (Figs. 1*E*,*F*, 6*A*,*E* ). Interestingly, our qPCR analysis showed that cocaine exposure minimally increased PARP-1 mRNA levels (Fig. 2*F*,*G*), similar to the observations by *Scobie et. al.* that induction of PARP-1 protein expression by cocaine is not entirely dependent on increase in mRNA levels (Scobie et al., 2014). These observations led us to hypothesize that cocaine-mediated downregulation of miR-125b is the key driver of the increased PARP-1 expression through post-transcriptional mechanism(s). Initial *in silico* analysis predicted a binding site of miR-125b in the 3’UTR sequence of PARP-1 that is conserved in several mammalian species (Fig. 3*A*,*B*). Genetic experiments demonstrated that altering miR-125b levels either by anti-miRs or miR-mimics negatively correlated with PARP-1 protein expression in neuronal cells (Fig. 3*C*,*D*). Subsequently, luciferase reporter based analysis revealed that miR-125b targets the 3’UTR region of PARP-1 mRNA (Fig. 4*A-D*). Finally, specific mutations in the 3’UTR of PARP-1 confirmed that miR-125b directly binds to PARP-1 3’UTR to inhibit protein translation (Fig. 4*E*). These results identify miR-125b as a key post-transcriptional/translational regulator of PARP-1 expression in neuronal cells.

Differential regulation of miRNAs in brain reward regions in response to cocaine has been reported by several laboratories (Chandrasekar and Dreyer, 2009; Eipper-Mains et al., 2011; Heyer and Kenny, 2015). Accordingly, we observed downregulation of miR-125b and induction of PARP-1 by cocaine in a region specific manner. Cocaine exposure reduced miR-125b levels and enhanced PARP-1 expression primarily in the NAc (Figs. 1*E*, 2*D*,*E* ) but not in the PFC (Fig. 8*A–C*). PARP-1 expression also remained unchanged in the VTA region in response to cocaine (Fig. 8*E*,*F*). However, increased levels of miR-125b were observed in VTA on cocaine treatment (Fig. 8*D*). The lack of negative correlation between miR-125b expression and PARP-1 levels in the VTA suggested alternative regulatory mechanism(s) in this region of the brain. It is well recognized that regionally selective gene expression patterns drive the behavioral changes involved in drug addiction and reward (Nestler, 2004). One of the hallmark effects of cocaine-induced behavioral changes are mediated by alteration in structure and function of medium spiny neurons (MSNs) and their inputs (Lee et al., 2006; Anderson and Self, 2017). These alterations are dependent on localized *de novo* protein synthesis at specific synapses that modulate dendrite morphology (Schratt et al., 2006). Cocaine-responsive miRNAs have been identified that could possibly regulate dendritic morphology and long-lasting cocaine mediated synaptic plasticity (Schratt et al., 2006; Magill et al., 2010; Giusti et al., 2014). For example, overexpression of miR-125b induced formation of long thin spines, while reduced miR-125b increases the width of dendritic spines. (Edbauer et al., 2010). Thus, reduced miR-125b levels in the NAc by cocaine treatment may regulate dendritic spine formation. In addition, PARP-1 expression in NAc after chronic cocaine administration was shown to modulate dendritic plasticity via targeting sidekick-1 (SDK1; Scobie et al., 2014; Scobie, 2015). Therefore, miR-125b alone or by upregulating PARP-1 expression may regulate dendritic spine structure that plays important role in the behavioral response to cocaine addiction.

The rewarding and reinforcing effects of cocaine are mediated primarily by dopaminergic neurotransmission (Kuhar et al., 1991). Cocaine inhibits reuptake of dopamine by binding to DAT (Beuming et al., 2008) resulting in an increased levels of extracellular dopamine that stimulates reward pathways in the brain (Kalivas, 2007). In addition, mice that express a cocaine insensitive DAT variant do not elevate extracellular dopamine levels as compared to wild type mice and do not exhibit cocaine-induced reward (O'Neill et al., 2014; Wu et al., 2014). Since DAT is central to cocaine action, we probed whether binding of cocaine to DAT is essential for the downregulation of miR-125b. To test this, we used the DAT blocker- nomifensine (Ferris et al., 2012). Nomifensine serves as an excellent probe to study DAT, since this compound binds to a pocket on DAT that is believed to be not targeted by cocaine (Tella et al., 1997). Due to this differential binding, the neuroadaptive molecular changes induced by nomifensine, have been suggested to cause limited abuse potential compared to cocaine (Tella et al., 1997; Jones et al., 1995). For example, up-regulation of DAT in caudate putamen and NAc was observed only with cocaine but not with nomifensine (Jones et al., 1995). This was also substantiated in our studies, wherein cocaine exposure resulted in the downregulation of miR-125b in DAT expressing cells while nomifensine had no effect (Fig. 5*D*). These properties of nomifensine enabled us to specifically demonstrate that binding of cocaine to DAT is critical for the downregulation of miR-125b. We observed that pretreatment of nomifensine to dopaminergic neuronal cells, abrogated both cocaine’s effect on miR-125b expression (Fig. 5*E*), and cocaine-induced enhancement of PARP-1 protein expression (Fig. 5*F*,*G*). We further confirmed the role of DAT in cocaine-induced downregulation of miR-125b, by using HEK-293T cells that stably express DAT and endogenously express miR-125b (Fig. 5*B*). Whether this dependence on DAT in a neuronal cell model will also be applicable *in vivo* and the DAT-dependent downregulation of miR-125b is dependent on increased levels of dopamine are currently being investigated. Nevertheless, it is plausible that the role of DAT on miR-125b/PARP-1 axis in the neuronal cells could be attributed to local variability in extracellular dopamine levels.

In conclusion, we have demonstrated a post-transcriptional mechanism of regulation of PARP-1 expression via miR-125b. The persistent downregulation of miR-125b in the NAc during acute and chronic cocaine treatment suggest the importance of this brain enriched miRNA in cocaine-induced response. Thus, understanding the biology of miR-125b may advance the cellular and molecular mechanisms of cocaine addiction and reward.

## Synthesis

Reviewing Editor: Leonidas Stefanis, Biomedical Research Foundation of the Academy of Athens Decisions are customarily a result of the Reviewing Editor and the peer reviewers coming together and discussing their recommendations until a consensus is reached. When revisions are invited, a fact-based synthesis statement explaining their decision and outlining what is needed to prepare a revision will be listed below. The following reviewer(s) agreed to reveal their identity: Lindsay De Biase

A major concern was the paucity of in vivo data. In order for us to reconsider the manuscript, you would have to provide time course and regional data showing the pattern of expression of miR125b and PARP-1 in different brain regions and at various time points following cocaine administration. Furthermore, you would need to address the concerns of the reviewers regarding statisctics, presentation of results, and overstatmenet of the results in the discussion section, as detailed in the reviewers' comments below.

Reviewer 1: The authors provide firm evidence that PARP-1 is post-transcriptionally regulated by miR125b using cell line experiments. Rodent studies strengthen the findings that both acute and 7 days of cocaine produce similar effects. The potential mechanism of action via 3'-UTR binding adds additional support. Whether these molecular changes are involved in the behavioral actions of cocaine is currently unknown and will be addressed in future studies. Overall, the studies are well designed and executed. The results add to our understanding of PARP1 regulation. However, the authors overstate their results and make claims that are unsupported. These points should be addressed adequately.

The authors mention in the significance section that due to their work PARP-1 can be used for therapeutic intervention in neurological disorders. This statement is very premature as they do not have any toxicity, behavioral, pharmacokinetic or pharmacodynamic data to support such a weighty claim.

What is the time course of PARP-1 protein induction in the animal studies? How many min/hours after drug treatment?

The author's claim that they provide evidence that miR125b is critical for cocaine's actions on the CNS (statement in Discussion), but no such experiment was performed by them. Was the miR manipulated in vivo, followed by testing to examine response to cocaine?

Minor: PARP-1 is spelled as PAPR-1 in Fig. 4 legend caption.

Reviewer 2:

1. The way in which qPCR data are quantified and displayed throughout the paper is confusing and inconsistent. Given that this is a key technical approach on which many of the paper's conclusions rely, the methods need to more clearly and extensively describe how data are quantified and the y-axes on all qPCR graphs need to be labeled with clear information about what is being displayed. For qPCR data, expression levels (Ct) of target gene are typically normalized to expression levels (Ct) of a housekeeping gene (GAPDH or similar, referred to as deltaCt). The relative expression level of the target gene across multiple samples can then be displayed as 2-deltaCt. In this case, the y-axis should be labeled "relative expression level, a.u." for arbitrary units. Instead, if one wants to calculate fold change of gene expression in treated samples compared to control, this is computed by subtracting deltaCt of treated from deltaCt of control and is then graphed as 2-delta delta Ct. In this case, the y-axis should be labeled "fold change treated over control" or similar. When displayed in this way, expression levels of untreated cells or saline injected animals should be 1 and cocaine treated cells or animals should have positive or negative fold changes in target gene expression relative to these controls.

The methods of the present manuscript would seem to indicate that all qPCR data are expressed as fold change (2-delta delta Ct of treated relative to control groups). However, in figure 1D,E, figure 3C,D, figure 5E, and figure 6A,E, graphs are shown as though fold changes have been calculated relative to cocaine treated samples. Or perhaps these are actually relative expression levels (2-deltaCt) but not appropriately labeled as such? Instead, in figure 2F,G and figure 6B, qPCR expression levels seem to be displayed as % of control value, with controls being 100% and treated cells or animals being a lower or higher %. If these are fold changes that are being displayed as % of control expression level, this is somewhat unconventional. Some of the qPCR graphs have "normalized" on the y-axis but it is unclear what is meant by normalized. In figure 6 - it is unclear what the difference is between A and B. They both show qPCR results of miR-125b levels in SH-SY5Y cells after chronic cocaine treatment but A shows "expression of treated cells relative to control" and B shows "fold change of treated cells relative to control from 3 independent experiments". This would seem to indicate that both are showing fold change. But perhaps A is showing "relative expression of miR-125b in control and cocaine-treated cells" (2-deltaCt) and B is showing "fold change of miR-125b expression in cocaine-treated cells over control" (2-delta delta Ct) but is graphed as % of control expression level?

2. It is not clear whether the appropriate statistical tests have been used to determine whether observed effects are significant. The authors need to more fully describe statistical approaches used in the methods and report statistical analyses more fully in the figure legends. The methods say that one way ANOVA was used to determine significance in dose dependent experiments (presumably data in figure 1D, 2F,C, 5B,C). However, ANOVA F and P values are not reported in the text or the figure legends. The asterisks and P-values reported in the figure legends appear to refer to specific individual comparisons presumably made with t-test (though this is not explicitly stated). But the methods do not describe what posthoc tests were performed after ANOVA and what approaches, if any, were used to correct for multiple comparisons. All of the experiments presented in the paper can be appropriately analyzed using one-way ANOVA or student's T-test. Yet, the methods state that aside from one-way ANOVA used in dose-response experiments, two-way ANOVA was used for other experiments. There don't appear to be any experiments in this manuscript in which analysis with two-way ANOVA would be appropriate.

3. miR-125b regulation of PARP-1 could be relevant for shaping gene expression and cellular status in a number of contexts and there are many ways in which the authors could expand our understanding of the functional relevance of this molecular interaction. In this manuscript, they choose to focus on whether this molecular interaction is relevant for cocaine-induced changes in PARP-1 levels. However, a causal link between miR-125b and cocaine-induced changes in PARP-1 levels in vivo is not demonstrated and they do not provide any evidence that miR-125b (or PARP-1) play a role in cocaine-induced changes in neuronal physiology or behavior. They instead refer to a single publication that indicates that PARP-1 increases in the NAc are relevant for cocaine-induced behaviors. If the authors are particularly intent on studying miR-125b/PARP-1 interactions in the context of cocaine exposure, they should provide some additional evidence that miR-125b plays a critical role in regulating PARP-1 changes induced by in vivo cocaine exposure. Some experiments that would strengthen the claim that miR-125b is relevant for understanding changes in neuronal function / behavior following in vivo cocaine exposure could be:

- Does overexpression of miR-125b in the NAc block cocaine's ability to increase PARP-1 expression? This could be accomplished using viral vectors and stereotaxic injection in the NAc. Such mice could also potentially be used to see if miR-125b overexpression alters basic cocaine induced behaviors such as locomotor sensitization or conditioned place preference. Alternatively, viral approaches could be used to knock-down miR-125b within the NAc and evaluate whether this leads to increases in PARP-1 protein and occlusion of cocaine's ability to increase PARP-1 in the NAc.

- In which cell types are cocaine-induced changes in miR-125b and PARP-1 taking place in vivo? Many of the hallmark cocaine-induced changes in behavior are mediated by alterations in the structure and function of medium spiny neurons and their inputs. Indeed, Scobie et. al 2014 provided evidence that PARP-1's role in cocaine-induced behavior changes was via alteration of MSN gene expression, leading to changes in dendritic spine structure. Can it be shown that in vivo cocaine exposure leads to miR-125b decreases and PARP-1 increases in MSNs? In situ hybridization approaches could be used to show that miR-125b is expressed in MSNs and decreased by in vivo cocaine exposure. Similarly, immunostaining approaches could be used to examine PARP-1 levels in MSNs following in vivo cocaine exposure. Alternatively, FACS-isolated MSNs could be used for qPCR quantification of miR-125 and, potentially, western blot experiments for PARP-1 after saline or cocaine injections. (One complication for this line of experimentation is that MSNs are not DAT-expressing cells, so the authors would need to re-evaluate whether cocaine binding to DAT is also required in vivo for downregulation of miR-125b).

- The time course over which cocaine-induced changes in MSN structure and function and over which cocaine-induced behaviors emerge are well described. How does this align with cocaine-induced changes in miR-125b in vivo? What is the time course and dose response of decreases in miR-125b in NAc that are induced by in vivo cocaine exposure? Is this phenomena observed over the same range of cocaine doses that are known to be relevant for behaviors such as locomotor sensitization and CPP? How soon after cocaine exposure are miR-125b decreases observed and how long do they last?

- How regionally specific are decreases in miR-125b that are induced by in vivo cocaine exposure? If miR-125b downregulation and ensuing PARP-1 upregulation play a key role in cocaine-induced changes in neuronal function and behavior, and these phenomena are dependent on cocaine binding to DAT, one might expect these changes to be much more prominent within nuclei of the mesolimbic dopamine system than in some comparison brain region where dopamine elevations and cocaine-induced changes in neuronal function are not as apparent.

4. The proposed mechanism that cocaine binding to DAT is required for miR-125b's effect on PARP-1 is complicated to interpret when applied to the in vivo setting. Scobie et al. 2014 indicate that PARP-1 contributes to cocaine-induced behaviors through binding to DNA and altering expression of target genes within the NAc. In the NAc, DAT expression is limited to the terminals of dopamine-producing neurons that project to this nucleus from the ventral tegmental area. If the model is that cocaine binding to DAT causes down-regulation of miR-125b and increases in PARP-1 only within the DAT-expressing cells, we would be expecting miR-125b/PARP-1 alterations in the DA neuron terminals within the NAc. Stable expression of miRNAs has been reported in axons and nerve terminals (see Natera-Naranjo 2010 RNA), but this doesn't explain how this would then lead to PARP-1 mediated changes in gene expression within neurons that reside in the NAc. On the other hand, if the model is that cocaine binding to DAT causes non-cell autonomous changes in miR-125b / PARP-1 expression (potentially through elevations in extracellular dopamine), one would expect any DAT blocker, including nomifensine, to lead to miR-125b downregulation in the NAc. The discussion needs to address how the authors envision that the molecular interactions they find in vitro will be taking place in vivo.

Minor concerns:

1. Different modes of cocaine administration can cause different cellular and molecular changes within mesolimbic dopamine circuits. What changes are observed also depend on what time point after drug administration is examined. For all in vivo experiments, it should always be specified in the text of the results or in the figure legends how cocaine was administered (i.p. or s.c. injection) and when tissue was collected following that injection (6 hours later? 24 hours later? 24 hours after the final injection?).

## References

[B1] Anderson EM, Self DW (2017) It's only a matter of time: longevity of cocaine-induced changes in dendritic spine density in the nucleus accumbens. Curr Opin Behav Sci 13:117–123. 10.1016/j.cobeha.2016.11.013 28607946 PMC5465866

[B2] Barr TL, Conley YP (2007) Poly(ADP-ribose) polymerase-1 and its clinical applications in brain injury. J Neurosci Nurs 39:278–284. 17966294 10.1097/01376517-200710000-00004

[B3] Bartel DP (2004) MicroRNAs: genomics, biogenesis, mechanism, and function. Cell 116:281–297. 14744438 10.1016/s0092-8674(04)00045-5

[B4] Bartel DP (2009) MicroRNAs: target recognition and regulatory functions. Cell 136:215–233. 10.1016/j.cell.2009.01.002 19167326 PMC3794896

[B5] Beuming T, Kniazeff J, Bergmann ML, Shi L, Gracia L, Raniszewska K, Newman AH, Javitch JA, Weinstein H, Gether U, Loland CJ (2008) The binding sites for cocaine and dopamine in the dopamine transporter overlap. Nat Neurosci 11:780–789. 10.1038/nn.2146 18568020 PMC2692229

[B6] Blaho K, Logan B, Winbery S, Park L, Schwilke E (2000) Blood cocaine and metabolite concentrations, clinical findings, and outcome of patients presenting to an ED. Am J Emerg Med 18:593–598. 10.1053/ajem.2000.9282 10999576

[B7] Chandrasekar V, Dreyer JL (2009) microRNAs miR-124, let-7d and miR-181a regulate cocaine-induced plasticity. Mol Cell Neurosci 42:350–362. 10.1016/j.mcn.2009.08.009 19703567

[B8] Chandrasekar V, Dreyer JL (2011) Regulation of MiR-124, Let-7d, and MiR-181a in the accumbens affects the expression, extinction, and reinstatement of cocaine-induced conditioned place preference. Neuropsychopharmacology 36:1149–1164. 10.1038/npp.2010.250 21307844 PMC3079833

[B9] Chao J, Nestler EJ (2004) Molecular neurobiology of drug addiction. Annu Rev Med 55:113–132. 10.1146/annurev.med.55.091902.103730 14746512

[B10] Chen CL, Liu H, Guan X (2013) Changes in microRNA expression profile in hippocampus during the acquisition and extinction of cocaine-induced conditioned place preference in rats. J Biomed Sci 20:96. 10.1186/1423-0127-20-96 24359524 PMC3878172

[B64] Chen S-M, Chou W-C, Hu L-Y, Hsiung C-N, Chu H-W, Huang Y-L, Hsu H-M, Yu J-C, Shen C-Y (2015) The Effect of MicroRNA-124 Overexpression on Anti-Tumor Drug Sensitivity. Gonzalez P, ed. PLoS ONE 10(6):e0128472. 10.1371/journal.pone.0128472 PMC4482746 26115122

[B11] Cohen-Armon M, Visochek L, Katzoff A, Levitan D, Susswein AJ, Klein R, Valbrun M, Schwartz JH (2004) Long-term memory requires polyADP-ribosylation. Science 304:1820–1822. 10.1126/science.1096775 15205535

[B12] Doetsch M, Gluch A, Poznanović G, Bode J, Vidaković M (2012) YY1-binding sites provide central switch functions in the PARP-1 gene expression network. PLoS One 7:e44125. 10.1371/journal.pone.0044125 22937159 PMC3429435

[B13] Doura MB, Unterwald EM (2016) MicroRNAs modulate interactions between stress and risk for cocaine addiction. Front Cell Neurosci 10:125. 10.3389/fncel.2016.00125 27303265 PMC4880569

[B14] Edbauer D, Neilson JR, Foster KA, Wang CF, Seeburg DP, Batterton MN, Tada T, Dolan BM, Sharp PA, Sheng M (2010) Regulation of synaptic structure and function by FMRP-associated microRNAs miR-125b and miR-132. Neuron 65:373–384. 10.1016/j.neuron.2010.01.005 20159450 PMC5018398

[B15] Eipper-Mains JE, Kiraly DD, Palakodeti D, Mains RE, Eipper BA, Graveley BR (2011) microRNA-Seq reveals cocaine-regulated expression of striatal microRNAs. RNA 17:1529–1543. 10.1261/rna.2775511 21708909 PMC3153976

[B16] Ferris MJ, Calipari ES, Mateo Y, Melchior JR, Roberts DC, Jones SR (2012) Cocaine self-administration produces pharmacodynamic tolerance: differential effects on the potency of dopamine transporter blockers, releasers, and methylphenidate. Neuropsychopharmacology 37:1708–1716. 10.1038/npp.2012.17 22395730 PMC3358740

[B17] Fontan-Lozano A, Suarez-Pereira I, Horrillo A, del-Pozo-Martin Y, Hmadcha A, Carrion AM (2010) Histone H1 poly[ADP]-ribosylation regulates the chromatin alterations required for learning consolidation. J Neurosci 30:13305–13313. 10.1523/JNEUROSCI.3010-10.2010 20926656 PMC6634736

[B18] Giusti SA, Vogl AM, Brockmann MM, Vercelli CA, Rein ML, Trumbach D, Wurst W, Cazalla D, Stein V, Deussing JM, Refojo D (2014) MicroRNA-9 controls dendritic development by targeting REST. Elife 3 10.7554/eLife.02755 PMC4235007 25406064

[B19] Ha HC, Snyder SH (2000) Poly(ADP-ribose) polymerase-1 in the nervous system. Neurobiol Dis 7:225–239. 10.1006/nbdi.2000.0324 10964595

[B20] Hartfield EM, Yamasaki-Mann M, Ribeiro Fernandes HJ, Vowles J, James WS, Cowley SA, Wade-Martins R (2014) Physiological characterisation of human iPS-derived dopaminergic neurons. PLoS One 9:e87388. 10.1371/journal.pone.0087388 24586273 PMC3931621

[B21] Hassa PO, Hottiger MO (1999) A role of poly (ADP-ribose) polymerase in NF-kappaB transcriptional activation. Biol Chem 380:953–959. 10.1515/BC.1999.118 10494847

[B22] Heard K, Palmer R, Zahniser NR (2008) Mechanisms of acute cocaine toxicity. Open Pharmacol J 2:70–78. 10.2174/1874143600802010070 19568322 PMC2703432

[B23] Heyer MP, Kenny PJ (2015) Corticostriatal microRNAs in addiction. Brain Res 1628:2–16. 10.1016/j.brainres.2015.07.047 26253823

[B24] Hollander JA, Im HI, Amelio AL, Kocerha J, Bali P, Lu Q, Willoughby D, Wahlestedt C, Conkright MD, Kenny PJ (2010) Striatal microRNA controls cocaine intake through CREB signalling. Nature 466:197–202. 10.1038/nature09202 20613834 PMC2916751

[B25] Im HI, Hollander JA, Bali P, Kenny PJ (2010) MeCP2 controls BDNF expression and cocaine intake through homeostatic interactions with microRNA-212. Nat Neurosci 13:1120–1127. 10.1038/nn.2615 20711185 PMC2928848

[B26] Jonas S, Izaurralde E (2015) Towards a molecular understanding of microRNA-mediated gene silencing. Nat Rev Genet 16:421–433. 10.1038/nrg3965 26077373

[B27] Jones SR, Garris PA, Wightman RM (1995) Different effects of cocaine and nomifensine on dopamine uptake in the caudate-putamen and nucleus accumbens. J Pharmacol Exp Ther 274:396–403. 7616424

[B28] Kalivas PW (2007) Neurobiology of cocaine addiction: implications for new pharmacotherapy. Am J Addict 16:71–78. 10.1080/10550490601184142 17453607

[B29] Karch SB, Stephens B, Ho CH (1998) Relating cocaine blood concentrations to toxicity–an autopsy study of 99 cases. J Forensic Sci 43:41–45. 10.1520/JFS16087J 9456523

[B30] Krishnakumar R, Kraus WL (2010) PARP-1 regulates chromatin structure and transcription through a KDM5B-dependent pathway. Mol Cell 39:736–749. 10.1016/j.molcel.2010.08.014 20832725 PMC2939044

[B31] Kuhar MJ, Ritz MC, Boja JW (1991) The dopamine hypothesis of the reinforcing properties of cocaine. Trends Neurosci 14:299–302. 1719677 10.1016/0166-2236(91)90141-g

[B32] Lagos-Quintana M, Rauhut R, Yalcin A, Meyer J, Lendeckel W, Tuschl T (2002) Identification of tissue-specific microRNAs from mouse. Curr Biol 12:735–739. 12007417 10.1016/s0960-9822(02)00809-6

[B33] Le MT, Teh C, Shyh-Chang N, Xie H, Zhou B, Korzh V, Lodish HF, Lim B (2009a) MicroRNA-125b is a novel negative regulator of p53. Genes Dev 23:862–876. 10.1101/gad.1767609 19293287 PMC2666337

[B34] Le MT, Xie H, Zhou B, Chia PH, Rizk P, Um M, Udolph G, Yang H, Lim B, Lodish HF (2009b) MicroRNA-125b promotes neuronal differentiation in human cells by repressing multiple targets. Mol Cell Biol 29:5290–5305. 10.1128/MCB.01694-08 19635812 PMC2747988

[B35] Lee KW, Kim Y, Kim AM, Helmin K, Nairn AC, Greengard P (2006) Cocaine-induced dendritic spine formation in D1 and D2 dopamine receptor-containing medium spiny neurons in nucleus accumbens. Proc Natl Acad Sci USA 103:3399–3404. 10.1073/pnas.0511244103 16492766 PMC1413917

[B36] Lin S, Gregory RI (2015) MicroRNA biogenesis pathways in cancer. Nat Rev Cancer 15:321–333. 10.1038/nrc3932 25998712 PMC4859809

[B37] Lupo B, Trusolino L (2014) Inhibition of poly(ADP-ribosyl)ation in cancer: old and new paradigms revisited. Biochim Biophys Acta 1846:201–215. 10.1016/j.bbcan.2014.07.004 25026313

[B38] Magill ST, Cambronne XA, Luikart BW, Lioy DT, Leighton BH, Westbrook GL, Mandel G, Goodman RH (2010) microRNA-132 regulates dendritic growth and arborization of newborn neurons in the adult hippocampus. Proc Natl Acad Sci USA 107:20382–20387. 10.1073/pnas.1015691107 21059906 PMC2996687

[B39] Mantri CK, Pandhare Dash J, Mantri JV, Dash CC (2012) Cocaine enhances HIV-1 replication in CD4+ T cells by down-regulating MiR-125b. PLoS One 7:e51387. 10.1371/journal.pone.0051387 23251514 PMC3520918

[B40] Meloche J, Le Guen M, Potus F, Vinck J, Ranchoux B, Johnson I, Antigny F, Tremblay E, Breuils-Bonnet S, Perros F, Provencher S, Bonnet S (2015) miR-223 reverses experimental pulmonary arterial hypertension. Am J Physiol Cell Physiol 309:C363–C372. 10.1152/ajpcell.00149.2015 26084306

[B41] Mittleman RE, Wetli CV (1984) Death caused by recreational cocaine use. An update. JAMA 252:1889–1893. 6471319

[B42] Morales J, Li L, Fattah FJ, Dong Y, Bey EA, Patel M, Gao J, Boothman DA (2014) Review of poly (ADP-ribose) polymerase (PARP) mechanisms of action and rationale for targeting in cancer and other diseases. Crit Rev Eukaryot Gene Expr 24:15–28. 10.1615/CritRevEukaryotGeneExpr.2013006875 24579667 PMC4806654

[B43] Nestler EJ (2004) Historical review: molecular and cellular mechanisms of opiate and cocaine addiction. Trends Pharmacol Sci 25:210–218. 10.1016/j.tips.2004.02.005 15063085

[B44] Nestler EJ (2004) Molecular mechanisms of drug addiction. Neuropharmacology 47 [Suppl 1]:24–32. 10.1016/j.neuropharm.2004.06.031 15464123

[B45] Oei SL, Griesenbeck J, Schweiger M, Babich V, Kropotov A, Tomilin N (1997) Interaction of the transcription factor YY1 with human poly(ADP-ribosyl) transferase. Biochem Biophys Res Commun 240:108–111. 10.1006/bbrc.1997.7621 9367892

[B46] O'Neill B, Tilley MR, Han DD, Thirtamara-Rajamani K, Hill ER, Bishop GA, Zhou FM, During MJ, Gu HH (2014) Behavior of knock-in mice with a cocaine-insensitive dopamine transporter after virogenetic restoration of cocaine sensitivity in the striatum. Neuropharmacology 79:626–633. 24412674 10.1016/j.neuropharm.2013.12.023 PMC4011184

[B47] Peretti FJ, Isenschmid DS, Levine B, Caplan YH, Smialek JE (1990) Cocaine fatality: an unexplained blood concentration in a fatal overdose. Forensic Sci Int 48:135–138. 10.1016/0379-0738(90)90105-8 2283136

[B48] Picciotto MR (2010) Neuroscience: microRNA knocks down cocaine. Nature 466:194–195. 10.1038/466194a 20613832

[B49] Rehmsmeier M, Steffen P, Höchsmann M, Giegerich R (2004) Fast and effective prediction of microRNA/target duplexes. RNA 10:1507–1517. 10.1261/rna.5248604 15383676 PMC1370637

[B50] Ritz MC, Lamb RJ, Goldberg SR, Kuhar MJ (1987) Cocaine receptors on dopamine transporters are related to self-administration of cocaine. Science 237:1219–1223. 2820058 10.1126/science.2820058

[B51] Schiewer MJ, Knudsen KE (2014) Transcriptional roles of PARP1 in cancer. Mol Cancer Res 12:1069–1080. 10.1158/1541-7786.MCR-13-0672 24916104 PMC4134958

[B52] Schratt GM, Tuebing F, Nigh EA, Kane CG, Sabatini ME, Kiebler M, Greenberg ME (2006) A brain-specific microRNA regulates dendritic spine development. Nature 439:283–289. 10.1038/nature04367 16421561

[B53] Scobie KN (2015) Poly(ADP)-ribose polymerase-1 inhibitors as a potential treatment for cocaine addiction. CNS Neurol Disord Drug Targets 14:727–730. 26022260 10.2174/1871527314666150529150013

[B54] Scobie KN, Damez-Werno D, Sun H, Shao N, Gancarz A, Panganiban CH, Dias C, Koo J, Caiafa P, Kaufman L, Neve RL, Dietz DM, Shen L, Nestler EJ (2014) Essential role of poly(ADP-ribosyl)ation in cocaine action. Proc Natl Acad Sci USA 111:2005–2010. 10.1073/pnas.1319703111 24449909 PMC3918779

[B55] Sievers F, Wilm Dineen AD, Gibson T, Karplus J, Li K, Lopez W, McWilliam R, Remmert H, Soding MJ, Thompson JD, Higgins DG (2011) Fast, scalable generation of high-quality protein multiple sequence alignments using Clustal Omega. Mol Syst Biol 7:539. 21988835 10.1038/msb.2011.75 PMC3261699

[B56] Tella SR, Ladenheim B, Cadet JL (1997) Differential regulation of dopamine transporter after chronic self-administration of bupropion and nomifensine. J Pharmacol Exp Ther 281:508–513. 9103538

[B57] Van Dyke C, Barash PG, Jatlow P, Byck R (1976) Cocaine: plasma concentrations after intranasal application in man. Science 191:859–861. 10.1126/science.56036 56036

[B58] Verdone L, La Fortezza M, Ciccarone F, Caiafa P, Zampieri M, Caserta M (2015) Poly(ADP-ribosyl)ation affects histone acetylation and transcription. PLoS One 10:e0144287. 10.1371/journal.pone.0144287 26636673 PMC4670112

[B59] Wang SH, Liao XM, Liu D, Hu J, Yin YY, Wang JZ, Zhu LQ (2012) NGF promotes long-term memory formation by activating poly(ADP-ribose)polymerase-1. Neuropharmacology 63:1085–1092. 10.1016/j.neuropharm.2012.06.050 22771769

[B60] Wei H, Yu X (2016) Functions of PARylation in DNA damage repair pathways. Genomics Proteomics Bioinformatics 14:131–139. 10.1016/j.gpb.2016.05.001 27240471 PMC4936651

[B61] Wu H, O'Neill B, Han DD, Thirtamara-Rajamani K, Wang Y, Gu HH (2014) Restoration of cocaine stimulation and reward by reintroducing wild type dopamine transporter in adult knock-in mice with a cocaine-insensitive dopamine transporter. Neuropharmacology 86:31–37. 10.1016/j.neuropharm.2014.04.022 24835281 PMC4188717

[B62] Xu LF, Wang J, Lv FB, Song Q (2013) Functions of microRNA in response to cocaine stimulation. Genet Mol Res 12:6160–6167. 10.4238/2013.December.4.2 24338410

[B63] Zaniolo K, Desnoyers S, Leclerc S, Guérin SL (2007) Regulation of poly(ADP-ribose) polymerase-1 (PARP-1) gene expression through the post-translational modification of Sp1: a nuclear target protein of PARP-1. BMC Mol Biol 8:96. 10.1186/1471-2199-8-96 17961220 PMC2175517

